# Response: “Commentary: Ultrasound-Guided Triamcinolone Acetonide Hydrodissection for Carpal Tunnel Syndrome: A Randomized Controlled Trial”

**DOI:** 10.3389/fmed.2022.841609

**Published:** 2022-03-08

**Authors:** Jia-Chi Wang, Po-Cheng Hsu, Kevin A. Wang, Ke-Vin Chang

**Affiliations:** ^1^Department of Physical Medicine and Rehabilitation, Taipei Veterans General Hospital, Taipei, Taiwan; ^2^School of Medicine, National Yang Ming Chiao Tung University, National Yang Ming University, Taipei, Taiwan; ^3^Division of General Surgery, Department of Surgery, Shin-Kong Memorial Hospital, Taipei, Taiwan; ^4^School of Medicine, Fu Jen Catholic University, New Taipei City, Taiwan; ^5^Department of Physical Medicine and Rehabilitation and Community and Geriatric Research Center, National Taiwan University Hospital, Bei-Hu Branch and National Taiwan University College of Medicine, Taipei, Taiwan; ^6^Center for Regional Anesthesia and Pain Medicine, Wang-Fang Hospital, Taipei Medical University, Taipei, Taiwan

**Keywords:** carpal tunnel syndrome, hydrodissection, median nerve, injection, corticosteroid

We appreciate the insightful comments (1) from Lam et al. regarding our study titled “Ultrasound-Guided Triamcinolone Acetonide Hydrodissection for Carpal Tunnel Syndrome: A Randomized Controlled Trial” (2) published in Frontiers in Medicine. The dose of triamcinolone acetonide for both groups should be 10 mg in accordance with the statement in ClinicalTrials.gov (Identifier: NCT04346030). We apologize for the misplacement of 40 mg/ml in the abstract.

Regarding the question, could the concentration of corticosteroid play a role in the clinical effect of hydro-dissection as the amount of the injectate was different between Group 1 and 2? Based on our antecedent randomized controlled trial (3), ultrasound-guided injection with 10 or 40 mg triamcinolone acetonide yielded similar improvements for patients with carpal tunnel syndrome. In the aforementioned study (3), the total volume of injectate was 2 ml in either the 10- or 40-mg group. Obviously, the 40-mg group received a higher concentration (and dose) of perineural corticosteroid than the 10-mg group. However, no additional benefit was observed in the 40-mg group, which might partially resolve the query from Dr. Lam et al.

Regarding the effect of hydrodissection, we are not sure whether the factor that Dr. Lam mentioned really contributed to the equal effectiveness in both groups. The formation of a halo can be derived from two clinical scenarios of perineural injections. First, the needle is introduced into the epineurium to let the fluid confine inside the nerve sheath like the common approach for sciatic nerve block. Second, if the target is the subsynovial connective tissue in the carpal tunnel, a circumferential shape of fluid accumulation can be formed by dissecting the upper and lower surfaces of the nerve with more fluid applied over the ulnar aspect. In Figure 1B of the commented article, there are some hyperechoic substances over the radial aspect of the median nerve. According to the anatomy and ultrasound appearance, we believe it to be the subsynovial connective tissues. Another point worth mentioning is that Figure 1B in the commented article was taken during the injection but not after the completion of the injection. Even so, if we carefully look at Figure 1B in the commented article, there is an area with mixed echogenicity radial to the median nerve. As the gain had been adjusted higher for counteracting the anisotropy (4) due to the wrist position, we believed that area had been infiltrated by the corticosteroid-containing injectate. Furthermore, the pathogenesis of carpal tunnel syndrome is complex and adhesion is only one of the proposed mechanisms. The aforementioned viewpoint is supported by our previous observational study (5), showing that the mobility of the median nerve was insignificantly affected by either corticosteroid injections or surgery although the substantial improvement was observed after both treatments. In this sense, more randomized controlled trials are needed to investigate the add-on effect of hydro-dissection considering the variation in the pharmacological effects of different regimens (6).

## Author Contributions

J-CW and K-VC: conceptualization, methodology, resources, funding acquisition, and formal analysis. P-CH: software. K-VC: investigation, supervision, and writing—review and editing. P-CH and KW: data curation and visualization. J-CW and KW: writing—original draft preparation. J-CW and P-CH: project administration. All authors contributed to the article and approved the submitted version.

## Conflict of Interest

The authors declare that the research was conducted in the absence of any commercial or financial relationships that could be construed as a potential conflict of interest.

## Publisher's Note

All claims expressed in this article are solely those of the authors and do not necessarily represent those of their affiliated organizations, or those of the publisher, the editors and the reviewers. Any product that may be evaluated in this article, or claim that may be made by its manufacturer, is not guaranteed or endorsed by the publisher.
